# Decrypting the phylogeny and metabolism of microbial dark matter in green and red Antarctic snow

**DOI:** 10.1093/ismeco/ycaf003

**Published:** 2025-01-10

**Authors:** Ze Ren, Wei Luo, Huirong Li, Haitao Ding, Yunlin Zhang

**Affiliations:** State Key Laboratory of Lake Science and Environment, Nanjing Institute of Geography and Limnology, Chinese Academy of Sciences, Nanjing 210008, China; University of Chinese Academy of Sciences (UCASNJ), Nanjing 211135, China; Key Laboratory for Polar Science, Polar Research Institute of China, Ministry of Natural Resources, Shanghai 200136, China; Antarctic Great Wall Ecology National Observation and Research Station, Polar Research Institute of China, Ministry of Natural Resources, Shanghai 200136, China; Key Laboratory of Polar Ecosystem and Climate Change, Shanghai JiaoTong University, Ministry of Education, Shanghai 200030, China; Key Laboratory for Polar Science, Polar Research Institute of China, Ministry of Natural Resources, Shanghai 200136, China; Antarctic Great Wall Ecology National Observation and Research Station, Polar Research Institute of China, Ministry of Natural Resources, Shanghai 200136, China; Key Laboratory of Polar Ecosystem and Climate Change, Shanghai JiaoTong University, Ministry of Education, Shanghai 200030, China; Key Laboratory for Polar Science, Polar Research Institute of China, Ministry of Natural Resources, Shanghai 200136, China; Antarctic Great Wall Ecology National Observation and Research Station, Polar Research Institute of China, Ministry of Natural Resources, Shanghai 200136, China; Key Laboratory of Polar Ecosystem and Climate Change, Shanghai JiaoTong University, Ministry of Education, Shanghai 200030, China; State Key Laboratory of Lake Science and Environment, Nanjing Institute of Geography and Limnology, Chinese Academy of Sciences, Nanjing 210008, China; University of Chinese Academy of Sciences (UCASNJ), Nanjing 211135, China; University of Chinese Academy of Science, Beijing 100049, China

**Keywords:** CAZymes, nitrogen cycling, snow microbiome, metagenome-assembled genomes, Antarctic

## Abstract

Antarctic snow harbors diverse microorganisms, including pigmented algae and bacteria, which create colored snow patches and influence global climate and biogeochemical cycles. However, the genomic diversity and metabolic potential of colored snow remain poorly understood. We conducted a genome-resolved study of microbiomes in colored snow from 13 patches (7 green and 6 red) on the Fildes Peninsula, Antarctica. Using metagenome assembly and binning, we reconstructed 223 metagenome-assembled genomes (MAGs), with 91% representing previously unexplored microbes. Green snow (GS) and red snow (RS) showed distinct MAGs profile, with *Polaromonas* and *Ferruginibacter* as the most abundant genera, respectively. GS exhibited higher alpha diversity with more unique and enriched MAGs, while RS showed greater variability with higher beta diversity. All MAGs contained genes encoding auxiliary activities (AAs), carbohydrate esterases (CEs), glycoside hydrolases (GHs), and glycosyl transferases (GTs), indicating microbial degradation of complex carbon substrates. The most abundant enzymes included GT2 (cellulose synthase), GT4 (sucrose synthase), CE1 (acetyl xylan esterase), GT41 (peptide beta-N-acetylglucosaminyltransferase), and CE10 (arylesterase). GS had a higher abundance of GTs, whereas RS was enriched in GHs. Furthermore, 56% of MAGs contained genes for inorganic nitrogen cycling, with 18 gene families involved in assimilatory nitrate reduction, dissimilatory nitrate reduction, and denitrification. Potential coupling of nitrogen cycling and carbohydrate metabolism was observed at both genome and community levels, suggesting close links between these pathways, particularly through nitrate reduction during carbohydrate degradation. This study enhances our understanding of microbial metabolic functions in polar ecosystems and highlights their roles in maintaining Antarctic ecological stability.

## Introduction

Snowfields are a vital component of the Antarctic environment and significantly influence the global climate system [1]. These snowfields support a diverse microbial community, including algae, bacteria, fungi, and archaea [2–4]. During spring and summer, pigmented microorganisms contribute to the formation of vividly colored snow patches—spanning from greens and reds, to oranges, pinks, and yellows—influenced by factors such as solar radiation, nutrient availability, and the presence of liquid water [5, 6]. These dynamic colors are primarily attributed to the presence of specialized snow algae, which adjust their pigmentation to shield against harsh solar irradiation during their life cycles [7, 8]. Colored snow patches, especially green snow (GS) and red snow (RS), are prevalent in polar and mid-latitude mountainous regions, notable for their striking hues and ecological significance [9, 10]. Their metabolic activities contribute to diverse biogeochemical processes, with extensive research highlighting the roles of snow algae and associated bacteria in carbon fixation, nutrient cycling, and their influence on snowmelt rates [1, 2, 4, 5, 10]. Studies predicted that climate warming will intensify the expansion and frequency of colored snow patches in polar and alpine regions [11, 12], which could further accelerate snowmelt rates by reducing surface albedo, potentially altering biogeochemical cycles [13, 14].

Colored snowpacks have been identified as a storage for nutrients and organic carbon [15]. Within polar microbial communities, snow algae act as primary producers, accounting for most of the biomass and producing substantial organic carbon [1, 9, 14, 16]. These microorganisms exhibit biochemical adaptations to their environment: GS communities are protein-rich, with high chlorophyll content and metabolites associated with nitrogen and amino acid metabolism, while RS communities are characterized by an abundance of lipids and carbohydrates and higher carotenoid content [5, 6]. The primary production by snow algae not only contributes to local carbon cycles but also supports a complex web of microbial life [17]. For instance, heterotrophic bacteria utilize extracellular hydrolytic enzymes to break down particulate organic carbon (POC) and high molecular weight dissolved organic carbon (DOC) into lower molecular weight compounds, facilitating their uptake and utilization within microbial communities [18, 19]. This enzymatic degradation is crucial for carbon remineralization, ensuring the continuity of energy flows and nutrient cycles in extreme environments. Despite Antarctica’s generally oligotrophic environment, snowfields often act as nitrogen hotspots with high nitrate concentrations [15]. This nitrate primarily derives from marine fauna [15, 20–22] and atmospheric deposition [23, 24], stimulating snow algae bloom. Diverse microbes are integral to carbon and nitrogen cycling in these snowfields. However, our knowledge of organic carbon and nitrogen metabolisms in Antarctic colored snowpacks remains limited, particularly with respect to previously unknown microbes.

Next-generation sequencing (NGS) technologies, particularly shotgun metagenomic sequencing, have emerged as essential tools for exploring microbial communities, especially when microbes are unculturable, enabling the reconstruction of metagenome-assembled genomes (MAGs) and providing deep insights into microbial 'dark matter' previously unstudied [25, 26]. However, the application of MAGs in studying Antarctic microbial communities, particularly those in colored snow, remains limited. While existing studies have provided snapshots of microbial diversity and some functional insights [2, 5, 27, 28], they have not explored metabolic potentials and ecological interactions at the genomic level.

The Fildes Peninsula, located south of King George Island in the South Shetland Islands, is characterized by seasonal and semipermanent snowfields that interact closely with the marine environment. In these coastal regions, colored snow patches play important roles by influencing melting runoff, supporting the food web, and contributing degradation products to the biogeochemical cycles of the coastal system [29]. In this study, we collected green snow and red snow samples on the Fildes Peninsula. This study aimed to use MAGs to explore the metabolic potentials of bacterial communities in green and red Antarctic snow, with a focus on their roles in carbohydrate and nitrogen metabolisms. Genes encoding carbohydrate-active enzymes (CAZymes) and nitrogen cycling were identified within metagenomes of GS and RS. This study enhances our understanding of microbial diversity and functionality in extreme environments and highlights the importance of microbial processes in sustaining the health and stability of the Antarctic ecosystem.

## Materials and methods

### Study area and field sampling

**Figure 1:**
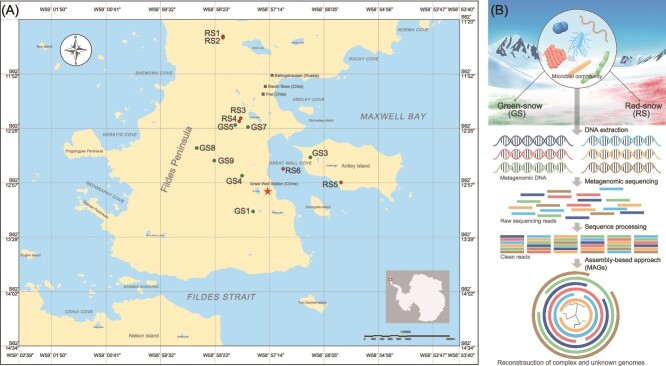
(A) GS and RS samples were collected on the Fildes Peninsula, King George Island, Antarctica. The red star represents the Great Wall Station of China. (B) A schematic process of metagenomic assembly.

The research was carried out on the Fildes Peninsula (Fig. 1A), located on King George Island, part of the South Shetland Islands in Antarctica. During December 2019 and January 2020, seven samples of GS and six samples of RS from the same location were meticulously gathered on the Fildes Peninsula (Fig. 1A). For consistency in sample collection, a standard sampling area of 50 × 50 cm^2^ snow patch was established at each site. The surface snow was carefully gathered into sterile plastic bottles, ensuring minimal contamination, and then transported back to the controlled environment of the laboratory at the Great Wall Station. The snow samples were then allowed to thaw naturally at a temperature of 4°C. Subsequently, 500 ml of the resulting meltwater was filtered using a 0.2-μm polycarbonate membrane filter (Whatman, UK) and stored at −80°C in a solution of cetyltrimethyl ammonium bromide until further analysis.

### DNA extraction, PCR, and metagenome assembly

Genomic DNA was extracted from the snow samples using the E.Z.N.A.® Soil DNA Kit (Omega Bio-tek, Norcross, GA, USA), following the manufacturer’s protocol. The quality and concentration of the extracted DNA were determined using the TBS-380 and NanoDrop2000 instruments, respectively. The integrity of the DNA was further verified by electrophoresis on a 1% agarose gel. For the construction of paired-end libraries, the DNA was fragmented to an average size of approximately 400 bp using the Covaris M220 instrument (Gene Company Limited, China). The NEXTflex Rapid DNA-Seq Kit (Bioo Scientific, Austin, TX, USA) was utilized to construct the libraries, with detailed procedures outlined in the supplementary materials. Paired-end sequencing was performed using the Illumina Hiseq Xten platform (Illumina Inc., San Diego, CA, USA) at Majorbio Bio-Pharm Technology Co., Ltd. (Shanghai, China) using HiSeq X Reagent Kits according to the manufacturer's guidelines (www.illumina.com).

Data analysis was executed on the Majorbio Cloud Platform (www.majorbio.com). Initially, Illumina paired-end reads were processed to remove adaptors and discard low-quality reads (those shorter than 50 bp, with a quality score below 20, or containing N bases) using fastp [30]. High-quality reads from each sample were individually assembled into contigs using MEGAHIT (v1.1.2) [31] set to default parameters within the MetaWRAP analysis workflow. Unmapped reads were removed. Binning of contigs was conducted using MetaBAT (v2.12.1) [32], and the bins were refined with the Bin_refinement module. The completeness, contamination, and strain heterogeneity of each bin were assessed using CheckM (v.1.0.12) [33]. Bins that met the criteria of ≥50% completeness, <10% contamination, and <10% strain heterogeneity were classified as MAGs. MAGs were dereplicated using dRep (v.3.4.2) with genomic average nucleotide identity (ANI) ≥99% and alignment coverage ≥10%, employing gANI as a secondary clustering method [34]. In instances where multiple MAGs corresponded to the same genomospecies, the highest quality MAG was selected as the representative for inclusion in the final MAG catalog and subsequent analyses. The abundance of MAGs was quantified using CoverM (https://github.com/wwood/CoverM, v0.6.1) as Reads Per Kilobase Million (RPKM). Taxonomic assignments of MAGs were performed using GTDB-Tk (v2.3.0) [35], and their gene predictions were performed using Glimmer [36]. The predicted genes were further annotated using Diamond against the Kyoto Encyclopedia of Genes and Genomes (KEGG) [37] and CAZymes [38] databases. Nitrogen cycling plays a critical role in altering the geochemical environment in snow patches. To infer the capacity for nitrogen cycling in these environments, MAGs were analyzed for metabolic genes involved in this process.

### Statistical analyses

The phylogenetic tree of all the reconstructed MAGs was built using the package *ggtree 3.8.2* [39]. The genomic map of the top three most abundant MAGs in GS and RS was created using the package *gggenes 0.5.1*, showing the distribution of genes associated with CAZymes and inorganic nitrogen cycling. Alpha diversity indexes, including species richness, Shannon diversity, and phylogenetic diversity, were calculated using the *vegan 2.6–4* package [40] and *picante 1.8.2* [41]. Beta diversity was calculated as the Bray–Curtis distance. Differences in alpha diversity and beta diversity between groups were assessed using the Wilcoxon-sum test. Nonmetric multidimensional scaling and adonis analyses were used to reveal the differences of MAG profiles and CAZymes composition between GS and RS. Heatmaps were used to visualize the relative abundance of certain MAGs and CAZyme families in colored snow samples. Co-occurrence patterns of nitrogen cycling genes and CAZyme families in GS and RS were analyzed, respectively, to reveal the possible coupling of nitrogen cycling and carbohydrate metabolism. Pearson correlation analysis was employed for co-occurrence assessment, with strong and statistically significant correlations included (*r* > 0.7 and *P* < .05). The co-occurrence networks were visually represented using *Gephi 0.1* [42]. All the statistical analyses were conducted in R 4.3.1 [43].

## Results

### MAG profiles in GS and RS

**Figure 2:**
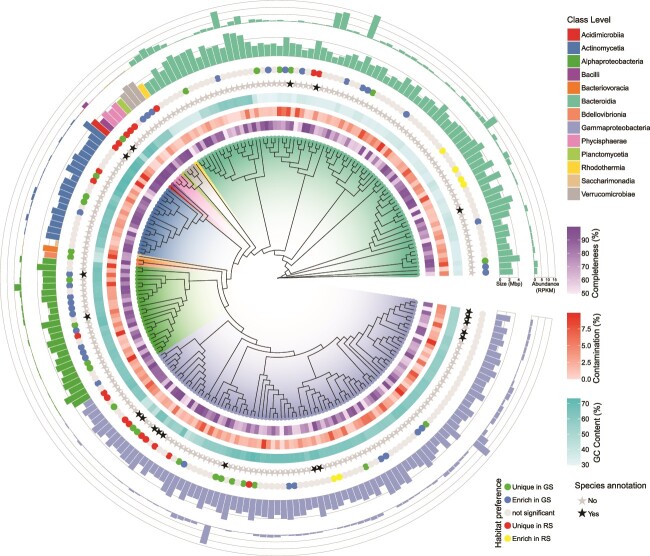
Phylogenetic tree of 223 reconstructed MAGs inferred from the GTDB based on conserved single-copy marker genes. The tree was colored at the class level. The rings from inner to outer represent completeness, contamination, GC content, species level annotation, habitat preference, size, and abundance of the corresponding MAG.

From GS and RS samples, the assembly and binning of metagenomic data yielded 223 MAGs (Fig. 2 and Table S1) with high completeness >50% (78.36% ± 14.53%) and low contamination <10% (3.07% ± 2.35%). Of the 223 MAGs, 58 MAGs were determined to be high quality, with the remaining 165 MAGs classified as medium quality. The extrapolated genome sizes ranged from 0.69 to 5.97 Mbp (3.23 ± 0.93 Mbp), with GC content ranging from 29.75% to 73.06% (52.56% ± 13.03%). The abundance of MAGs ranged from 0.006 to 17.17 rpkm (1.43 ± 2.33 rpkm), reflecting a broad spectrum of microbial representation, from extremely rare taxa to highly abundant ones, across the samples.

**Figure 3:**
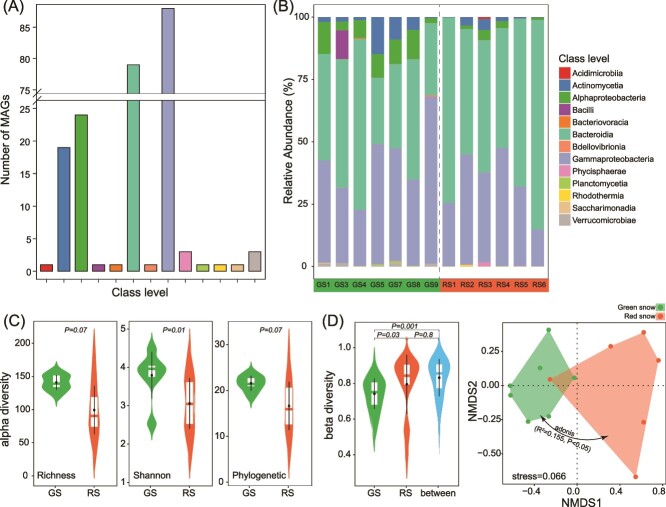
The composition and diversity of MAGs in GS and RS. (A) Number of MAGs at class level. (B) Relative abundance of classes across the samples. (C) Differences of alpha-diversity indexes between GS and RS. (D) Beta diversity and nonmetric multidimensional scaling of MAG profiles in GS and RS. Differences between groups were assessed using the Wilcoxon-sum test.

A maximum likelihood phylogenetic tree inferred from the GDTB provided a well-supported topology of the relative evolutionary relationships of these MAGs (Fig. 2). All reconstructed MAGs were identified as bacteria, belonging to 8 phylum and 13 classes (Fig. 2), with Gammaproteobacteria, Bacteroidia, Alphaproteobacteria, and Actinomycetia having a high number of MAGs (88, 79, 24, and 19 MAGs, respectively) (Fig. 3A). The classes Gammaproteobacteria, Bacteroidia, Alphaproteobacteria, and Actinomycetia were dominant in both GS and RS, with Bacteroidia and Gammaproteobacteria showing the highest relative abundance in GS (42.9% and 41.3%, respectively) and RS (62.9% and 34.8%, respectively) (Fig. 3B). There were 216 MAGs identified to genus level, but only 20 MAGs were identified to species level, indicating a large proportion (91%) of previously unexplored microbial taxa (Fig. 2). The most prevalent and abundant MAG in GS was an unclassified species in the genus *Polaromonas* (MAG146, Fig. S1), while in RS was an unclassified species in the genus *Pelobium* (MAG203, Fig. S1).

The composition of MAGs was different between GS and RS (Fig. 3). Among the reconstructed MAGs, 27 MAGs were only presented in GS, while 20 MAGs only presented in RS (Fig. S2), 176 MAGs were shared by GS and RS, and 23 MAGs were presented across all samples (Fig. 2). The most prevalent MAGs, which were presented in all samples, mainly consisted of the genera *Polaromonas, Aquabacterium_A, Hymenobacter, Halpernia, Pelobium, Ferruginibacter, Arcicella,* and *CAILRJ01* (Fig. S1). According to Wilcoxon test, the abundance of shared MAGs in the snow metagenome was compared between different color patches. For the shared 176 MAGs, 33 were significantly enriched in GS, while only 6 were enriched in RS (Figs 2 and S2). The results indicated a higher number of MAGs enriched in GS compared to RS, which could reflect differences in environmental conditions, resource availability, or other ecological factors influencing microbial community composition. Alpha-diversity analysis revealed an overall higher species richness, Shannon diversity, and phylogenetic diversity for MAGs in GS than in RS (Fig. 3C). However, RS samples exhibited higher beta diversity, indicating greater variation in microbial community composition between samples compared to GS, which showed more consistent community structures (Fig. 3D). NMDS and adonis (*R*^2^ = 0.155, *P* < .05) analyses showed that GS and RS had significantly different MAG compositions (Fig. 3D).

### Metabolic potential of complex carbohydrate metabolism

**Figure 4:**
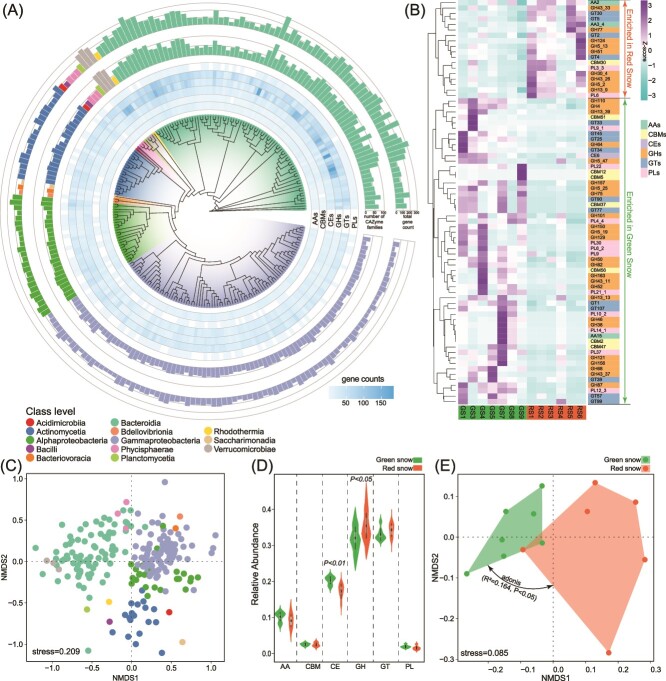
The profile of CAZymes in GS and RS. (A) Gene counts in each MAG. (B) Heatmap of CAZyme families significantly enriched in GS and RS. (C) Nonmetric multidimensional scaling of MAGs based on CAZyme families. (D) Relative abundance of genes encoding CAZyme classes. (E) Differences of CAZyme composition between GS and RS revealed by nonmetric multidimensional scaling, according to CAZyme families in each snow sample.

Within the snow metagenomes, our study targeted a comprehensive set of CAZymes, comprising 394 enzyme families (Table S2), including glycoside hydrolases (GHs, *n* = 203), glycosyl transferases (GTs, *n* = 69), polysaccharide lyases (PLs, *n* = 52), carbohydrate-binding modules (CBMs, *n* = 35), auxiliary activities (AAs, *n* = 19), and carbohydrate esterases (CEs, *n* = 16). These enzymes play critical roles in carbon cycling and the degradation of polysaccharides such as pectin, glycan, and glucan, which are commonly found in algal biomass and are likely present in the snow algae communities based on existing literature [5, 44, 45]. All the MAGs contained the genes encoding AAs, CEs, GHs, and GTs (Fig. 4A). Overall, the most prevalent genes across the MAGs were those encoding GTs (41 genes per MAG), followed by GHs (39.3 genes per MAG), CEs (23.6 genes per MAG), and AAs (11.7 genes per MAG) (Fig. 4A). However, the NMDS plot exhibited that these MAGs were taxonomically clustered based on the annotation of CAZyme genes (Fig. 4C). MAGs of Gammaproteobacteria, Bacteroidia, Alphaproteobacteria, and Actinomycetia were independently clustered (Fig. 4C), suggesting distinct carbohydrate degradation of different taxonomic groups. With respect to CAZymes genes, MAG75 (*Spirosoma*), MAG88 (*Ferruginibacter*), MAG198 (*Pelobium*), MAG176 (*Ferruginibacter*), and MAG195 (*Persicitalea*) were the top five MAGs containing the highest gene counts associated with CAZyems (Fig. 4A), particularly those genes encoding enzymes involved in degrading glycogen, polyphenol, cellulose, pectin, alpha-mannan, and glycan, the polysaccharides associated with microbial and algal biomass. These results elucidated the degradation of complex carbon substrates by microorganisms in colored snow.

A comparative study between GS and RS revealed significant differences in CAZymes composition. Based on the relative abundance of CAZymes families, NMDS and adonis (*R*^2^ = 0.164, *P* < .05) analyses showed clear differences between metagenomes in GS and RS, underlining the distinct microbial potential for carbohydrate degradation in different colored snow patches (Fig. 4E). In terms of relative abundance of associated genes, GTs (33%) were the most abundant in GS, followed by GHs (32%), CEs (20%), and AAs (10%) (Fig. 4D). While in RS, GHs (35%) were the most abundant, followed by GTs (34%), CEs (17%), and AAs (9%). GS exhibited higher abundance of CEs but lower GHs compared to RS (Fig. 4D). At the family level, GT2 (cellulose synthase: EC 2.4.1.12), GT4 (sucrose synthase: EC 2.4.1.13), CE1 (acetyl xylan esterase: EC 3.1.1.72), GT41 (peptide beta-N-acetylglucosaminyltransferase: EC 2.4.1.225), and CE10 (arylesterase: EC 3.1.1.2) presented the top five highest relative abundances in both GS and RS. In specific, 74 CAZymes families show significant differences between GS and RS (Fig. 4B). GS was enriched with 56 CAZymes families (25 GHs, 11 GTs, 11 PLs, 7 CBMs, 1 AA, and 1 CE), which mainly degradate glycan, glucan, pectin, carrageenan, xylan, chitin, and chitosan. However, RS showed enrichment in 18 CAZymes families (9 GHs, 4 GTs, 2 PLs, 2 AAs, and 1 CBM), which mainly degradate glucan, cellulose, lignin, and starch.

### Nitrogen cycling

**Figure 5:**
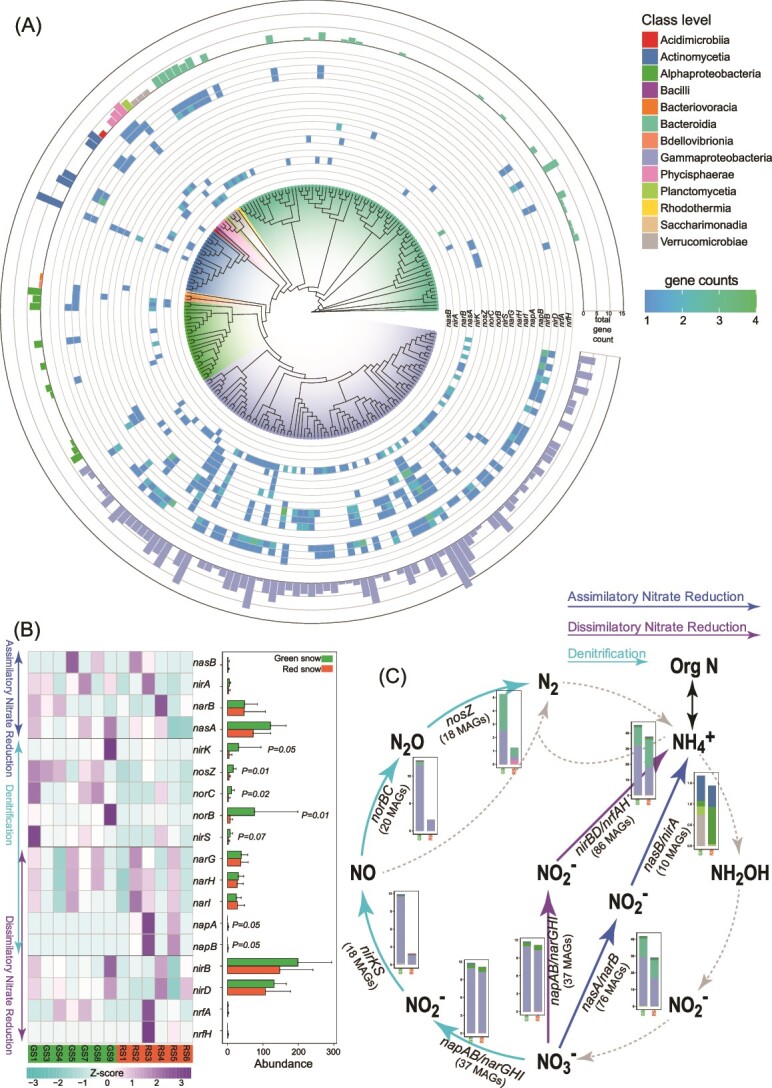
The profile of inorganic nitrogen cycling genes in GS and RS. (A) Gene counts in each MAG. (B) Gene abundance in GS and RS samples. (C) Nitrogen cycling pathways and associated genes and MAGs.

Across the reconstructed 223 MAGs, only 126 MAGs had the genes encoding inorganic nitrogen cycling, and only 18 gene families were predicted (Table S3), encoding assimilatory nitrate reduction (ANRA), dissimilatory nitrate reduction (DNRA), and denitrification (Fig. 5). However, genes involved in anaerobic ammonium oxidation, nitrogen fixation, and nitrification were not found.

In ANRA in the colored snow, the reduction of NO_3_^−^ to NO_2_^−^ was catalyzed by *nasA/narB*, and the reduction of NO_2_^−^ to NH_3_/NH_4_^+^ was catalyzed by *nasB*/*nirA* (Fig. 5A, C). There were 35% of MAGs contained these ANRA genes, indicating the potential for microbial assimilatory nitrogen reduction in the colored snow (Fig. 5A, C). However, only seven MAGs (mainly Actinomycetia) contained the genes catalyzing the complete pathways of ANRA. In DNRA in the colored snow, 45% of MAGs possessed *narGHI*/*napAB* (catalyze the reduction of NO_3_^−^ to NO_2_^−^) and *nirBD*/*nrfAH* (catalyze the reduction of NO_2_^−^ to NH_3_/NH_4_^+^) (Fig. 5A, C), suggesting that these microbes had a potential to use nitrate as electro acceptor. There were 22 MAGs (mainly Gammaproteobacteria) contained the genes catalyzing the complete pathways of DNRA. Moreover, in the process of denitrification, the reduction of NO_3_^−^ to NO_2_^−^ was catalyzed by *narGHI* and *napAB*, the reduction of NO_2_^−^ to NO was catalyzed by *nirKS*, NO to N_2_O was catalyzed by *norBC*, and the final step N_2_O to N_2_ was catalyzed by *nosZ* (Fig. 5C). Approximately 26% of MAGs contained denitrification genes. Seven organisms within Gammaproteobacteria (*Rhodoferax; Marinobacter; Thiobacillus; Janthinobacterium; Undibacterium; Rhodoferax;* and *Marinobacter*), most of which were enriched in GS samples, contained genes involved in the complete denitrification pathways of reducing NO_3_^−^ to N_2_, releasing the nitrogen compound back into the atmosphere.

### Potential coupling of carbohydrate metabolism and nitrogen cycling

The analysis of the top three most abundant MAGs from GS and RS revealed distinct genetic potentials for nitrogen cycling and carbohydrate metabolism between the two types of snow (Figs S3 and S4). In GS, the top MAGs contained genes associated with ANRA (*nasA*), DNRA (*nirBD*), and denitrification (*nirK, norB*), indicating their capacity to convert NO_3_^−^ to NO_2_^−^, NH_4_^+^, and N_2_ (Figs S3 and S4). Meanwhile, these MAGs also contained genes encoding a variety of CAZymes (Figs S3 and S4). Notably, CE1 enzymes (CE) were dominant with the highest gene count. CE1 catalyzes the breakdown of polysaccharides and other carbon-rich compounds, such as triacylglycerol, polyhydroxybutyrate, peptide, and S-formylglutathione (Figs S3 and S4). These findings suggested that the most abundant MAGs in GS play a significant role in nitrate reduction and removal, coupled with the metabolism of breaking down a wide range of complex organic materials. In contrast, the top MAGs from RS lacked genes for nitrogen cycling, with one MAG only containing the *nirBD* (Figs S3 and S4), indicating a limited capability for nitrogen transformations. However, these top MAGs in RS possessed a more diverse array of CAZymes, with the highest gene counts encoding GT2_Glycos_transf_2 (GTs), which are critical for glycosylation processes, including the formation of glycosylated products from activated sugars, as well as the formation of biofilm (Figs S3 and S4).

**Figure 6:**
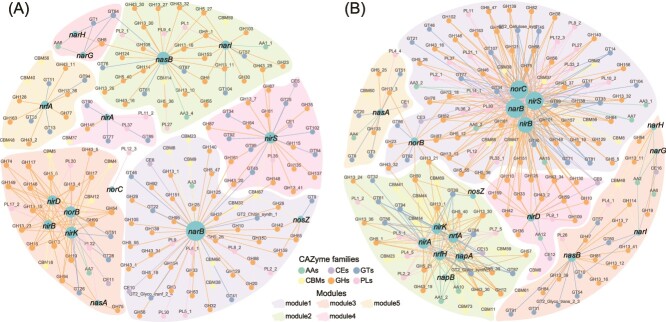
Co-occurrence of nitrogen cycling genes and CAZyme families in (A) GS and (B) RS. The note is colored according to CAZyme families. The polygon represents network module.

Beyond the distribution of genes within individual genomes, the co-occurrence patterns of nitrogen cycling genes and CAZyme families in GS and RS provided further insights into the possible coupling of N cycling and carbohydrate metabolism at the community level (Fig. 6). The network revealed extensive co-occurrence of nitrogen cycling genes (*narB, nirBD, nirKS, norBC*, etc) with various CAZyme families (Fig. 6). These findings highlighted the integrated interactions between nitrogen transformations and carbohydrate degradation processes in the snow microbial communities, despite 45% of MAGs and some dominant MAGs lacking nitrogen cycling genes (Fig. 5A). Some nitrogen cycling genes were closely connected to specific groups of CAZymes within distinct modules (Figs 6 and S5), suggesting specificities in the coupling between nitrogen cycling and carbohydrate metabolism. GS had more specialized couplings, whereas RS exhibited a more complex coupling network. These differential couplings underline the unique ecological roles and biogeochemical impacts of microbial communities in green and red Antarctic snow.

## Discussion

Analysis of MAGs from Antarctic GS and RS revealed distinct microbial communities with profound implications for ecological function and biogeochemical processes in these unique polar ecosystems. Our study indicated a significant untapped diversity, with 91% of MAGs not identified to the species level, suggesting a rich reservoir of genetic material and metabolic capabilities yet to be explored, commonly termed microbial 'dark matter' [46, 47]. Such vast unexplored diversity underscores the complexity and adaptability of microbial life to the extreme conditions of Antarctic snowfields. In GS, the predominant MAG was an unclassified species of *Polaromonas*, while in RS, the predominant MAG was an unclassified species of *Pelobium*. *Polaromonas,* the most abundant genus in our study, has been reported to play an essential role in the sustained growth of snow algae [4, 48]. In contrast, *Pelobium,* a less-studied genus [49], is thought to degrade polysaccharides and exhibit metabolic adaptations to cold temperatures and low ammonium availability. GS harbors more unique and enriched MAGs, along with higher alpha-diversity compared to RS, suggesting that GS environments might offer a broader range of ecological niches [2, 27]. Conversely, the higher beta diversity in RS indicates greater dissimilarity among its communities [27]. The adaptability of microbial communities to distinct snow types likely reflects their specialized functions in biogeochemical processes, such as their different capacities to degrade carbohydrates and utilize or transform available nitrogen.

As global temperatures rise and colored snow patches become more prevalent, biogeochemical processes in Antarctic snowfields are expected to become more important [9, 11, 50]. These processes could have cascading effects on broader biogeochemical cycles across these fragile ecosystems, including associated terrestrial and marine environments [6, 7, 13]. Snowpacks have been recognized as reservoirs for nutrients, DOC, and pigments [15]. Our findings suggest that microbial communities in these snowpacks possess a diverse array of genes involved in carbohydrate metabolism and organic matter degradation, contributing to C- and N cycling in Antarctic snowfields. Colored snow patches are rich in various organic compounds, including algae-derived biomass (e.g. lipids and fatty acids, starch, alginates, proteins, hemicellulose, carbohydrate esters, pectin, and lignin), pigments (e.g. chlorophyll, carotenoids, and astaxanthin), fungal-derived biomass (e.g. chitin and glucan), and bacteriaderived biomass (e.g. peptidoglycan) [5, 6, 51–53]. The decomposition of these organic matters is driven by CAZymes synthesized by bacteria [38, 54, 55]. Our metagenomic analysis revealed a diverse array of CAZymes in microbial communities from both GS and RS, highlighting their pivotal role in the biodegradation of complex carbohydrates such as pectin, glycan, and glucan. Notably, the distinct CAZymes profile observed in GS and RS suggests that microbial communities might be highly adapted to their specific microenvironments, possibly influenced by differences in organic matter compositions and physical conditions. These adaptations reflect distinct metabolic strategies employed by microbial communities in colored snow patches. For example, in GS, the high level of CEs and GTs suggests a more intensive breakdown and remodeling of algae-derived polysaccharides, likely driven by the higher organic matter content resulting from algal blooms. In contrast, RS exhibited higher levels of GHs, suggesting a more significant involvement in the degradation of cellulose and lignin-like compounds. Microbial communities in GS have access to more labile organic matter, which is easier to degrade than the organic carbon found in RS. The varied expression of CAZymes underscores the diverse carbohydrate components in these environments and the adaptability of microbial communities to their microenvironments [56, 57], enabling them to optimize metabolic processes according to available substrates and environmental pressures.

Antarctic colored snowfields generally have higher nitrate (NO_3_^−^) and phosphate concentrations compared to inland areas [15]. Marine fauna, including the feces from seal haul-outs, penguin colonies, and bird nesting sites, significantly contribute to nutrient hotspots in the typically nutrient-poor Antarctic environment, supplying essential sources of nitrogen and phosphate [15, 20–22]. Additionally, nitrogen deposition through precipitation serves as another key source of fixed nitrogen in snowfields [23, 58]. In these environments, NO_3_^−^ levels are influenced by microbial-driven processes, including ANRA and DNRA. Our results revealed the prevalence of genes associated with ANRA, DNRA, and denitrification, while genes for anaerobic ammonium oxidation, nitrogen fixation, and nitrification, aligning with the high NO_3_^−^ concentration in Antarctic colored snow [15]. In the assimilatory process, nitrate is reduced to ammonia, which is then incorporated into organic compounds [59]. A significant proportion (35%) MAGs contained ANRA genes (*nasA/narB* and *nasB/nirA*) in the colored snow, enabling the conversion of inorganic nitrogen into biologically useful forms. Conversely, DNRA reduces nitrate to ammonia without incorporating it into biomass [59]. DNRA is particularly critical in environments where nitrate serves as a preferred electron acceptor. In our study, DNRA genes (*narGHI*/*napAB* and *nirBD*/*nrfAH*) were prevalent and closely linked with carbohydrate degradation processes, both within a single genome and at the community level. When microbes degrade complex carbohydrates in colored snow, they generate electrons that require transfer to an electron acceptor to sustain metabolism, with nitrate serving as an ideal electron acceptor [60]. Moreover, the presence of denitrification genes (*narGHI/napAB, nirKS, norBC,* and *nosZ*) in colored snow indicated that microbes can utilize electrons derived from carbohydrate degradation to drive this pathway, effectively removing excess nitrogen from snowfield ecosystems, thereby regulating nitrogen levels and mitigating potential nitrogen-induced environmental impacts following snowmelt.

The interdependence of carbon and nitrogen cycles is a fundamental aspect of ecosystem dynamics. Our study demonstrated the potential coupling of CAZyme-associated reactions and nitrate reduction in colored snow, supported by evidences from both single-genome analyses and community-level co-occurrence networks. In GS, diverse genes encoding CAZymes and nitrogen cycling processes were identified within single genomes of the most abundant MAGs, indicating a direct coupling of carbohydrate metabolism and nitrate reduction within individual microorganisms. Conversely, the dominant MAGs in RS lacked such coupling within single genomes. However, the strong co-occurrence between nitrogen cycling genes and CAZyme families at the community level suggested that these metabolisms might be coupled in a more complex and distributed manner. GS exhibited more specialized couplings between nitrogen cycling and carbohydrate metabolism, while RS displayed a broader and more intricate coupling network, reflecting a wider range of metabolic interactions. The strong coupling of carbon and nitrogen metabolism highlights the intricate metabolic interdependencies within the microbial communities [61, 62], facilitating the efficient cycling of these elements and influencing energy flow within these ecosystems [63, 64]. While CAZymes themselves are not directly involved in using nitrate as an electron acceptor, the metabolic processes they initiate can produce intermediates (like pyruvate or acetate) that are further processed in pathways where nitrate reduction occurs. CAZymes break down complex carbohydrates into simpler sugars, which are subsequently metabolized to produce reduced cofactors such as NADH and FADH2, donating electrons for various redox reactions, including nitrate reduction. These differential coupling strategies observed in GS and RS suggest unique ecological roles and biogeochemical impacts of microbial communities in Antarctic snow, emphasizing the importance of understanding these interactions for broader ecological studies.

## Conclusions

This study utilized MAGs to investigate the metabolic potentials of bacterial communities in green and red Antarctic snow from the Fildes Peninsula. A total of 223 MAGs were reconstructed, with 91% remaining unclassified at the species level, representing a significant reservoir of microbial dark matter. Our findings demonstrate distinct microbial communities in GS and RS, with GS exhibiting higher alpha diversity and a greater abundance of nitrogen-cycling genes, while RS showed higher beta diversity and an enrichment of glycoside hydrolases. The potential coupling of carbohydrate metabolism and nitrogen cycling, observed at both the single-genome and community levels, suggests a close linkage between these metabolic pathways, with nitrate reduction serving as an electron acceptor during carbohydrate degradation. These findings highlighted the adaptability of microbial communities to their specific ecological niches and their critical roles in carbon and nitrogen cycling in Antarctic snowfields. The study underscores the importance of microbial dark matter in maintaining the ecological stability of these extreme environments and provides a foundation for future research on microbial adaptation and resilience in the face of global environmental changes. Future research should also focus on elucidating the metabolic roles of snow algae and their interactions with bacterial communities, as well as the broader impacts of microbial activity on global biogeochemical cycles.

## Supplementary Material

MS_SI_20240519_ycaf003

MS_SI_tables_20240519_ycaf003

## Data Availability

The raw sequence data reported in this paper have been deposited in the Genome Sequence Archive in National Genomics Data Center, China National Center for Bioinformation/Beijing Institute of Genomics, Chinese Academy of Sciences. The accession number is CRA014730, that are publicly accessible at https://ngdc.cncb.ac.cn/gsa/browse/CRA014730.
