# Supplementary material for: Decrypting the phylogeny and metabolism of microbial dark matter in green and red Antarctic snow
Source: ISME Commun. 2025 Jan 10;5(1):ycaf003. doi: 10.1093/ismeco/ycaf003 (PMC11765414; doi:10.1093/ismeco/ycaf003)

Supplementary Materials

Figure S1 Heatmap of 23 MAGs presented across all samples.

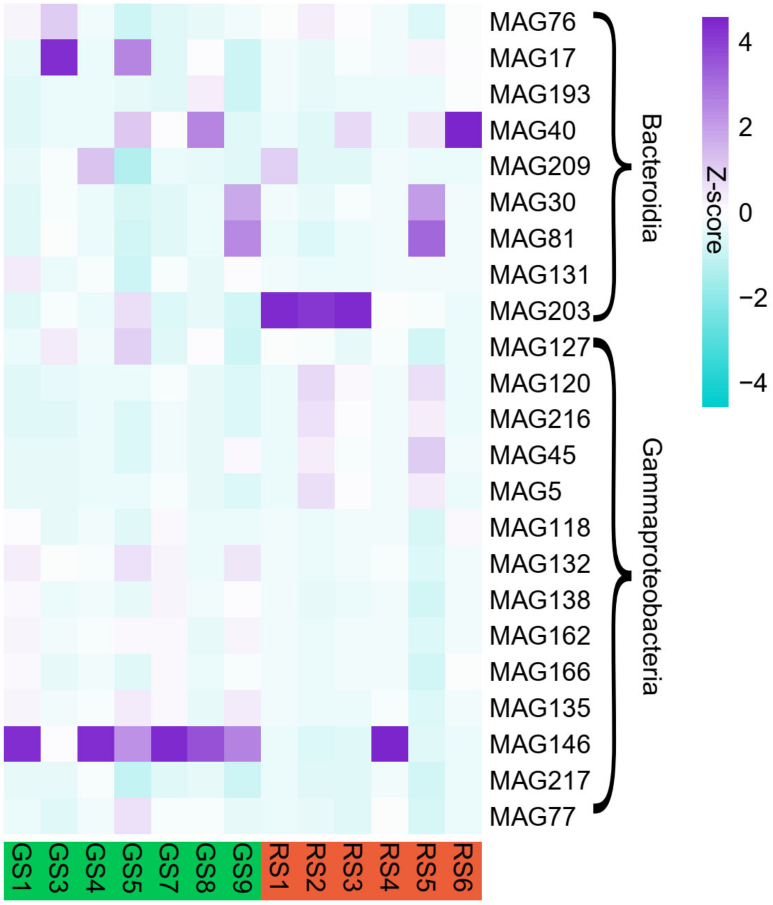

Figure S2 MAGs unique or enriched in green snow and red snow.

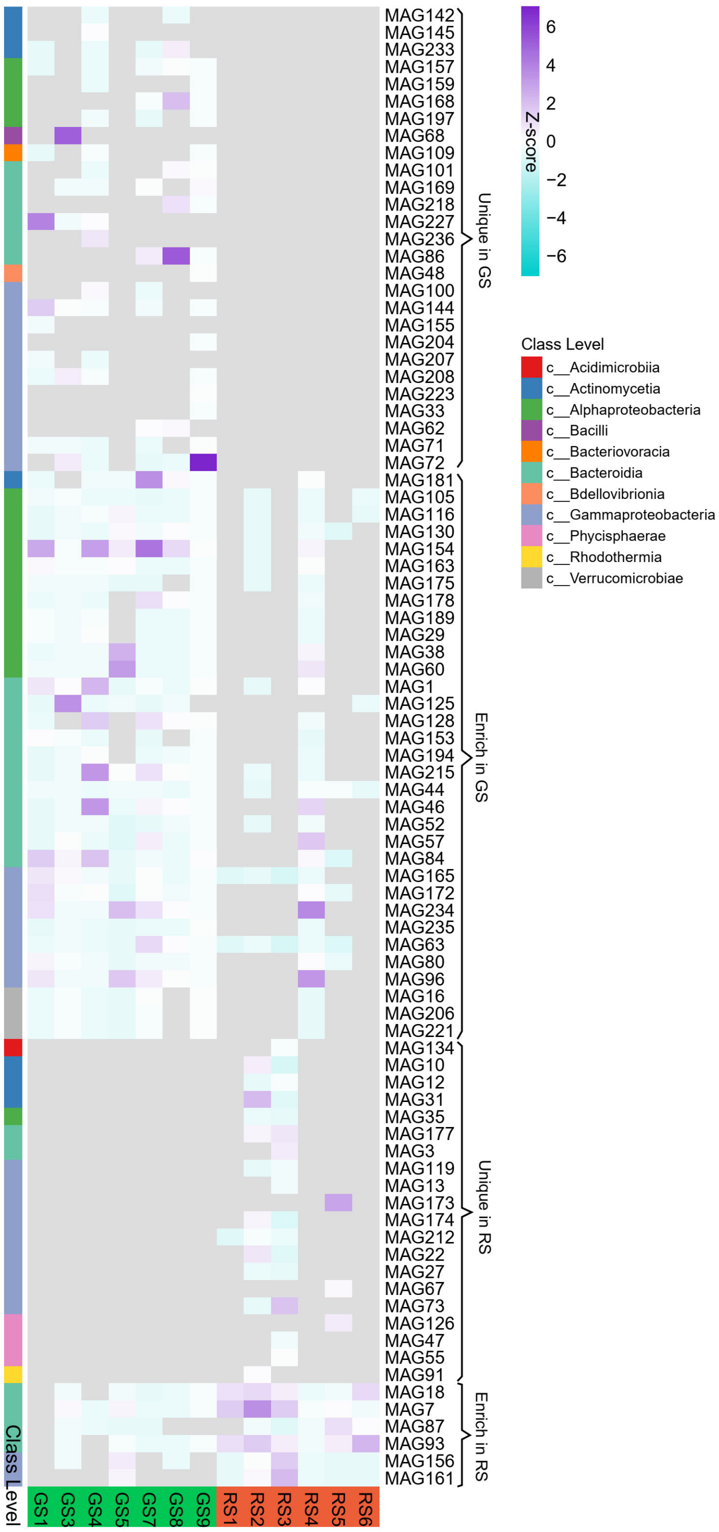

Figure S3 Nitrogen cycling genes and CAZyme families for the top 3 most abundant MAGs in (a) green snow and (b) red snow. The genus and species taxonomic information are shown. The pie plot represents the composition of CAZyme families for each MAG. The right panel represents the CAZyme with the highest gene count contained in each MAG, as well as the possible reactions catalyzed by this CAZymes.

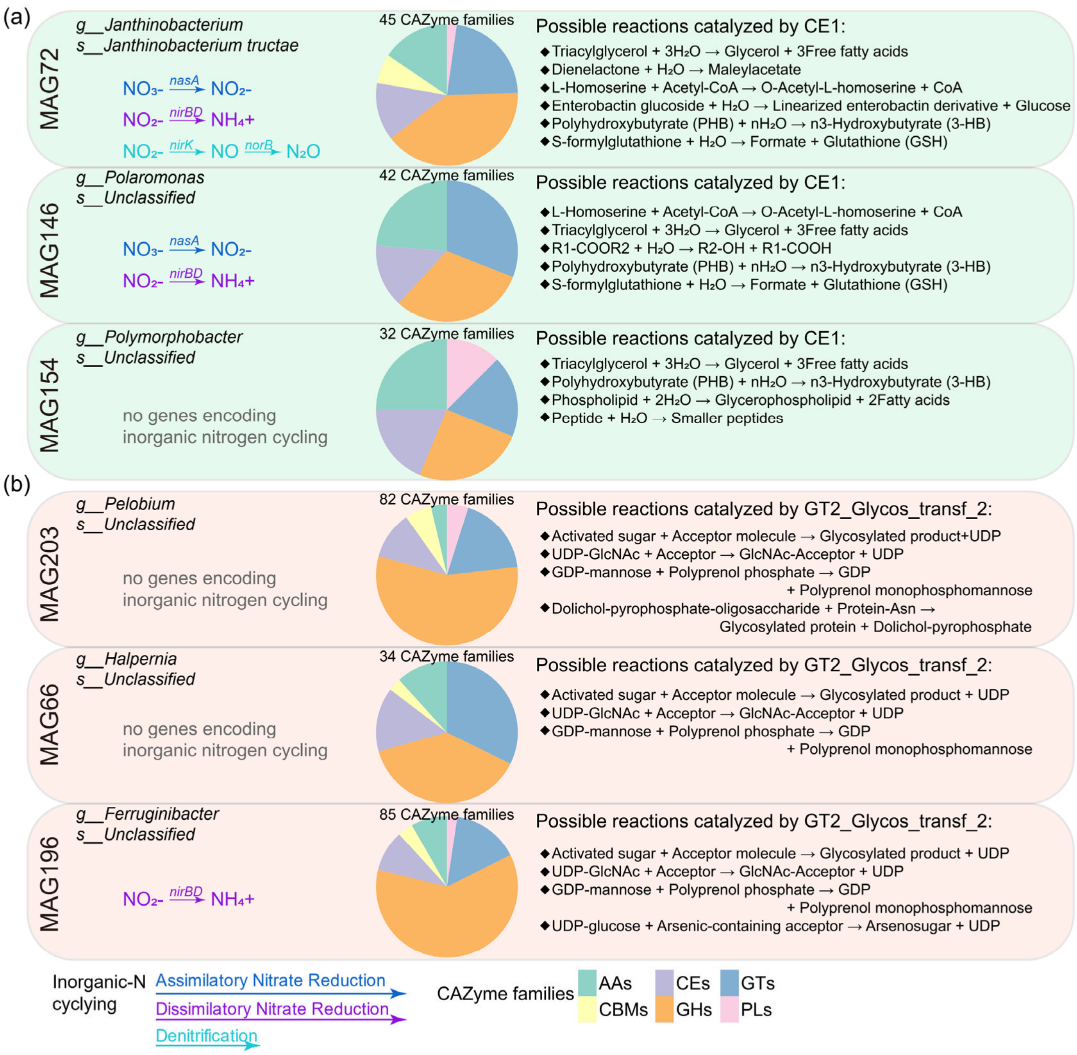

Figure S4 Genetic map of the scaffold encoding genes associated to CAZymes and inorganic nitrogen cycling for the top three most abundant MAGs in (a) green snow and (b) red snow.

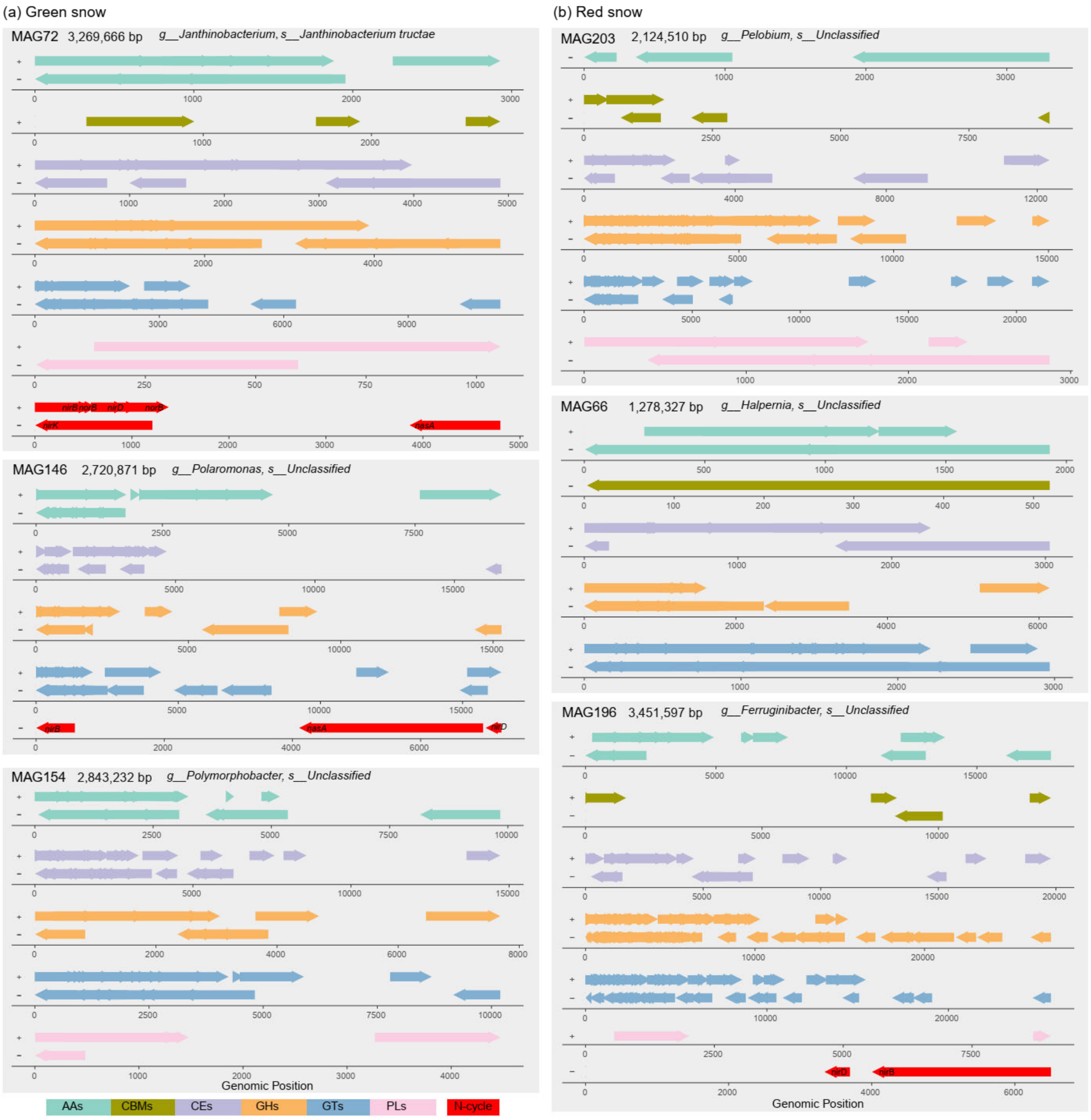

Figure S5 Modules in the co-occurrence of nitrogen cycling genes and CAZyme families in (a) green snow and (b) red snow. The bottom panel shown the composition of CAZyme families of each module in pie plots. The nitrogen cycling pathways encoding by the genes within each module were shown below the pie plot.

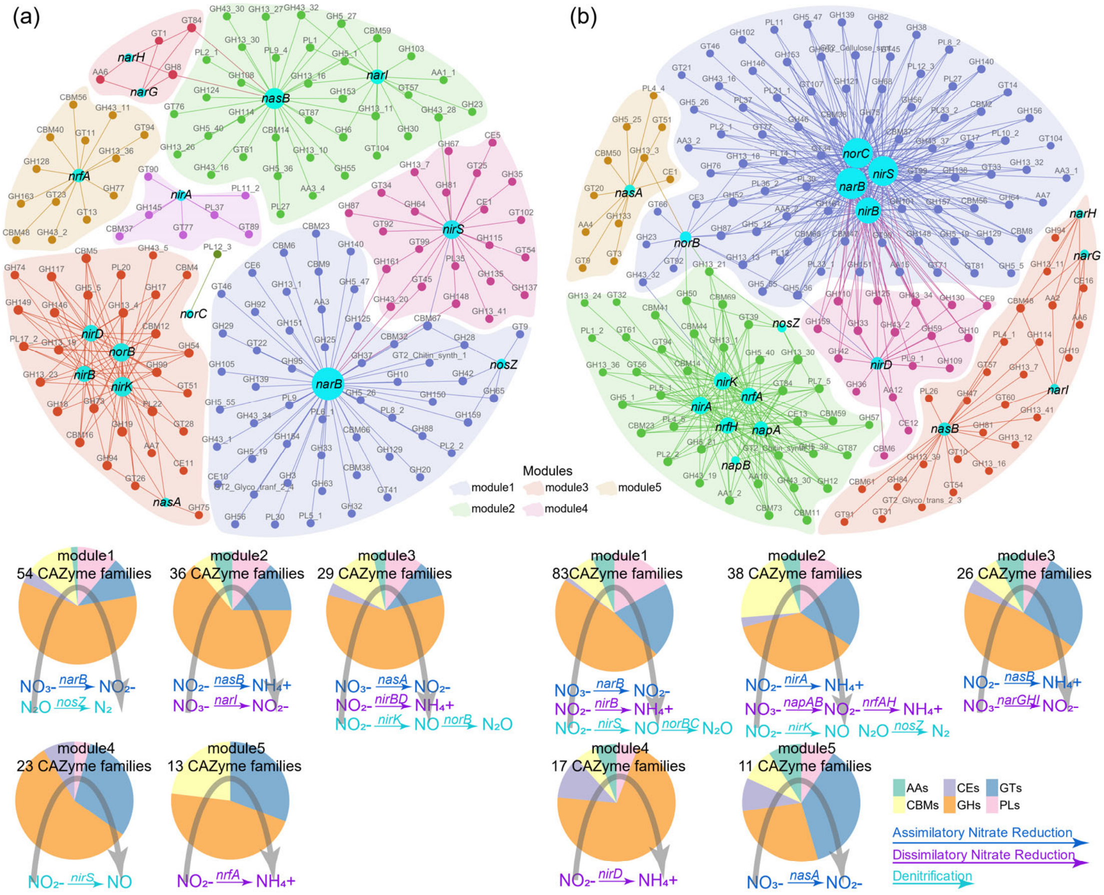

Supplement: MS_SI_20240519_ycaf003 [file ms_si_20240519_ycaf003.pdf]
